# Esophageal Mucosal Resistance in Reflux Esophagitis: What We Have Learned So Far and What Remains to Be Learned

**DOI:** 10.3390/diagnostics13162664

**Published:** 2023-08-12

**Authors:** Igor V. Maev, Maria A. Livzan, Sergei I. Mozgovoi, Olga V. Gaus, Dmitry S. Bordin

**Affiliations:** 1 Department of Propaedeutic of Internal Diseases and Gastroenterology, A.I. Yevdokimov Moscow State University of Medicine and Dentistry, 127473 Moscow, Russia; 2 Department of Internal Medicine and Gastroenterology, Omsk State Medical University, 644099 Omsk, Russia; 3 Department of Pathological Anatomy, Omsk State Medical University, 644099 Omsk, Russia; 4 Department of Pancreatic, Biliary and Upper Digestive Tract Disorders, A.S. Loginov Moscow Clinical Scientific Center, 111123 Moscow, Russia; 5 Department of Outpatient Therapy and Family Medicine, Tver State Medical University, 170100 Tver, Russia

**Keywords:** gastroesophageal reflux disease, mucosal resistance, reflux esophagitis, mucosal barrier

## Abstract

Gastroesophageal reflux disease (GERD) has the highest prevalence among diseases of the digestive system and is characterized by a significant decrease in patients’ quality of life, comparable to arterial hypertension and coronary heart disease. One in every ten cases of reflux esophagitis leads to the formation of Barrett’s esophagus, which is associated with a high risk of esophagus adenocarcinoma. The key factors determining the progression of the disease are the frequency and duration of the reflux of the stomach’s contents. As a result, refluxate, which includes hydrochloric acid, pepsin, and, in the case of concomitant duodeno-gastric reflux, bile acids and lysolecithin, is thrown into the overlying sections of the digestive tract. At the same time, in addition to aggression factors, it is necessary to take into account the state of resistance in the esophageal mucosa to the effects of aggressive refluxate molecules. This review was prepared using systematized data on the protective properties of the esophageal mucosa and modern methods to assess the mucosal barrier in reflux esophagitis. Lesions of the epithelial barrier structure in the esophagus are recognized as the main pathogenetic factor in the development of reflux esophagitis and are a potentially significant therapeutic target in the treatment of GERD and Barrett’s esophagus. This article presents the characteristics of the esophageal mucosal barrier and the protective mechanisms of the esophagus’s mucous membrane in conditions of gastroesophageal reflux. Diagnostic approaches for assessing the course of reflux esophagitis are described for both histological criteria and the possibility of a comprehensive assessment of the state of mucins, tight-junction proteins, and the proliferative activity of the mucosa, including under the conditions of ongoing therapy.

## 1. Introduction

Gastroesophageal reflux disease (GERD) has the highest prevalence among acid-dependent diseases [1,2] and, according to the Montreal Consensus, is defined as a condition that develops when gastric contents enter the esophagus, causing characteristic symptoms and/or complications [3].

The key factors determining the progression of the disease are the frequency and duration of the reflux of the stomach’s contents, as a result of which refluxate—which includes hydrochloric acid, pepsin, and, in the case of concomitant duodeno-gastric reflux, bile acids and lysolecithin—is thrown into overlying sections of the digestive tract [1,4,5]. The most pronounced destructive effect on the epithelium is exerted by gastric juice with unconjugated bile acids at pH values ranging from 1 to 3; therefore, the presence of bile refluxate significantly increases the risk of developing Barrett’s esophagus, dysplasia, and neoplasia of the esophageal epithelium [6,7]. It has been established that the synergism of hydrochloric acid, pepsin, and bile acids has the greatest damaging effect on the mucosa of the esophagus. The mucosa of the esophagus, when exposed to mixed reflux, is potentially damaged due to the negative chemical effect on the esophageal wall, as in classical gastroesophageal reflux, and also by a change in the composition of the esophageal microbiota under the action of bile acids [8,9].

Exposure to acid and bile salts stimulates the secretion of pro-inflammatory cytokines by epithelial cells, in particular interleukins-1, 6, 8, 10, and tumor necrosis factor-alpha, increasing the presence of T cells and neutrophils in the tissue with the development of a chronic active inflammatory process [10]. The combination of inflammatory processes, oxidative stress, and increased proliferative activity create a favorable environment for the formation of epithelial metaplasia (Barrett’s esophagus) with the subsequent progression of structural changes in the mucosa to adenocarcinoma of the esophagus [10,11].

When considering the mechanisms of formation and progression of reflux esophagitis, it is necessary to take into account, in addition to factors of aggression, the state of resistance of the esophagus’s mucosa to the effects of aggressive refluxate molecules. The violation of the structure of the esophagus’s epithelial barrier is recognized as the main pathogenetic factor in the development of reflux esophagitis and is a potentially significant therapeutic target in the treatment of GERD and Barrett’s esophagus [12,13]. Thus, the formation of reflux esophagitis (non-erosive and erosive of varying severity) and the complicated course of the disease (Barrett’s esophagus) occur with an imbalance between aggression and defense factors, and patients with lower esophageal resistance to reflux have a more severe reflux in esophagitis [14].

This review was conducted to systematize data on the protective properties of the esophageal mucosa and modern methods for assessing the mucosal barrier in reflux esophagitis.

A systematic search of articles was carried out in the PubMed/MEDLINE, Embase, and Google Scholar databases. Keyword combinations based on the medical subject heading (MeSH) were used to search for articles, including “gastroesophageal reflux disease”, “reflux esophagitis”, “mucosal barrier”, and “esophageal mucosal resistance”. According to the selection criteria, we analyzed full-text articles published in English from May 1998 up to May 2023, including original studies, systematic reviews, and meta-analyses. The authors independently reviewed and analyzed the articles. After applying the selection criteria, 59 articles were included in the final list of references.

## 2. Components of the Mucosal Barrier That Provide Resistance to the Esophageal Mucosa in Conditions of GERD

The most superficial, pre-epithelial level of protection is the mucus layer, which neutralizes the incoming acid and protects the squamous epithelium of the esophagus from contact with the reflux content [15]. Its key components are mucins, bicarbonates and non-bicarbonate buffers, prostaglandin E2, epidermal growth factor, and transforming growth factor alpha [16]. The main protective glycoproteins, mucins, enter the esophagus with saliva and are secreted by the esophagus’s glands. Mucins are present in a secreted form, which forms a protective layer over the epithelium, and in a form associated with the cell membrane, which is part of the glycocalyx and localized on the surface of epithelial cells [15]. It has been established that aggressive molecules in the composition of the refluxate, primarily hydrochloric acid, stimulate the secretion of mucins MUC3 and MUC5AC, while an increase in the secretion of mucins is associated with the restoration of the protective properties of the mucous membrane and vice versa; this is significantly reduced under conditions of the progression of esophagitis [17].

The resident microbiota are also commonly referred to as pre-epithelial protection factors, although their population is significantly smaller in comparison with other parts of the digestive tract [18]. The presence of data on the dominance of streptococci and the frequent presence of other taxa typical of the microbiota of the oropharynx are associated with the composition of the microflora of the oral cavity and pharynx, where a high prevalence of streptococci is found, along with taxonomic units, such as *Veillonella*, *Fusobacterium*, *Gemella*, *Granulicatella*, and *Rothia*, indicating that the microbiota of the esophagus are mainly of oral origin [19,20]. However, not all bacteria associated with the oral mucosa can colonize the esophageal mucosa, while some members of the esophageal microbiota are absent or present in small numbers in typical oral microflora, indicating the existence of microbiota. The esophagus is a separate microbiological ecosystem [21].

The next stage of protection is the mucosa of the esophagus proper (epithelial level), represented by the stratified squamous non-keratinized epithelium, which consists of three different layers: the surface layer of squamous epithelium cells, cells of the spiny layer, and the layer of basal cells [22]. The basal layer is usually represented by 1–3 layers of cells and comprises immature cells with relatively large nuclei and a relatively small amount of cytoplasm. These cells are the source of renewal for the epithelial layer, and the only cells in the esophageal epithelium that are capable of dividing with the subsequent migration of daughter cells towards the upper layers of the epithelium. During the process of such migration, the nuclei decrease in size, and the cell eventually enters the layer of superficially located mature cells [23]. In addition to the compact arrangement of cells, the presence of a layer of intercellular glycocalyx also provides additional protection against the penetration of aggressive refluxate components [24].

However, the most important mechanism for the formation of the integrity of the epithelial layer involves a special molecular complex that ensures the formation of cell contacts in the superficial and spinous layer. This structure, which forms intercellular contacts and regulates the diameter of the intercellular space, is known as the apical junction complex and includes three main components: tight-junction proteins, intercellular adhesion proteins, and desmosomes [25].

Tight-junction proteins play an important role in the formation of intercellular tight-junction complexes and are represented by occludins (OCLN), zonulins, adhesion junction molecules (JAM-A, JAM-B, JAM-C), and claudins, mainly claudin-nom-1 (CLDN1), claudin-2 (CLDN2), and claudin-4 (CLDN4). Claudins and occludins are transmembrane proteins and bind to cytoskeletal proteins (actin) through intracellular proteins, including zonulins ZO-1, ZO-2, and cingulin. Tight junctions make it possible to form a physical barrier that regulates the penetration of electrolytes and water between cells and prevents the penetration of bacteria and toxins through the epithelium [26,27,28]. The most significant tight-junction proteins in the mucosa of the esophagus are claudin-1 and claudin-4 [29].

Intercellular adhesion proteins provide the structural integrity of the epithelial lining by binding epithelial cells to each other.

Desmosomes in the stratified squamous epithelium not only isolate cells, but also carry out protein and ion transport through intercellular spaces. Desmosomes are represented by desmosomal cadherins with intercellular and extracellular domains that regulate the rate of ion exchange, proliferation, and polarization of epithelial cells [26,27,30].

The post-epithelial level of protection is provided by the blood supply to the mucosa and the mechanisms for maintaining the acid–base state of the tissue [16]. Thus, ionic H^+^-transporters are basolaterally located in the cell membranes of the esophageal epithelium and are able to remove excess H^+^ ions, increasing the cellular pH to normal values [15,31]. The mucosal bloodstream, in addition to providing nutrients and oxygen, delivers bicarbonates to the tissue and removes metabolic by-products, including hydrogen ions, lactic acid, and CO_2_. A number of studies have shown a compensatory increase in the blood supply to the esophageal mucosa when it is exposed to hydrochloric acid [22].

## 3. Methods for Diagnosing Reflux Esophagitis and Assessing the Resistance of the Esophageal Mucosa

The interaction between the aggressive properties of refluxate and the protective properties of the mucosa in the esophagus under conditions of pathological gastroesophageal reflux causes the formation of reflux esophagitis, which can be assessed both at the macroscopic level via endoscopy and at the microscopic level by examining biopsies of the esophageal mucosa.

During esophagogastroscopy, the severity of reflux esophagitis is assessed in accordance with the Los Angeles classification [3]. Identification of reflux esophagitis grade S-D is one of the most convincing diagnostic signs of GERD [32].

In addition to routine esophagogastroscopy, from the standpoint of assessing the stability of the esophageal mucosa under conditions of pathological gastroesophageal reflux, the novel method of endoscopic basic impedancemetry (BI) can be used. This method involves determining the basic impedance of the mucosa of the esophagus at rest in the absence of swallowing or reflux phenomena. Farré et al., based on in vivo and in vitro studies of acid perfusion in animals and humans, suggested that BI correlates with transepithelial resistance, a known marker of esophageal mucosal integrity [33]. It was found that patients with GERD (both erosive and non-erosive) had lower BI values compared with functional heartburn, which can serve as a reliable biomarker for GERD. The relationship between BI and common markers of GERD was further studied, and an association between reflux with long-duration acid exposure and DIS with low BI was found [34]. Subsequently, Savarino E. et al. confirmed the correlation between BI and histopathological markers of GERD, noting a lower BI in patients with erosive and non-erosive GERD compared with patients with functional heartburn [35].

Taking a biopsy and histological examination of biopsies of the mucous membrane of the esophagus is currently not a mandatory method for examining a patient suffering from GERD. Nevertheless, when performing an endoscopy, a biopsy should be taken in patients with a discrepancy between clinical and endoscopic data, with refractory GERD (lack of clinical and endoscopic remission within 1–2 months of treatment with a standard dose of PPI), an atypical course of erosive esophagitis, suspicion of the formation of Barrett’s esophagus, or the presence of neoplasms. High-resolution endoscopy (HD), narrow band endoscopy (NBI), and magnifying endoscopy can detect metaplastic and dysplastic areas of the esophageal epithelium and be used for targeted biopsy [36,37,38].

On the one hand, damage resulting from the contact of the mucous membrane with acid stimulates an increase in the proliferative potential and a significant thickening of the cell layer of the basal layer of the epithelium, i.e., basal cell hyperplasia. On the other hand, exposure to acid and bile salts stimulates the secretion of pro-inflammatory cytokines via epithelial cells, in particular interleukins-1, 6, 8, and 10, and tumor necrosis factor-alpha, increasing the presence of T-cells and neutrophils in the tissue [39]. Pro-inflammatory cytokines released by epithelial cells not only increase damage to the latter, but also activate mesenchymal and endothelial cells, stimulating the production of even more inflammatory mediators, with the involvement of immune cells and the formation of a vicious circle [10,22,40]. Reactive oxygen species released by immune cells react with the surrounding proteins and fatty acids of cell membranes, causing lipid peroxidation with the development of oxidative stress. Damage to the apical junction complexes and a decrease in the expression of tight-junction proteins, specifically claudin-1, claudin-2, claudin-4, zonulin, occludin, and cell adhesion proteins, together with the expansion of intercellular spaces in patients with GERD, along with additional factors that reduce epithelial protection, allow aggressive refluxate molecules to penetrate into the deeper layers of the mucosa [22,41].

A combination of basal cell hyperplasia, increased length of the papillae, intraepithelial inflammation, intercellular oedema with dilated intercellular spaces (spongiosis), balloon cells, and vascular changes in the squamous mucosa comprises the classical set of signs for a reflux pattern of injury. Because the histologic features are not specific, a number of additional features must be assessed before a definitive diagnosis of reflux esophagitis can be made.

The three major features of reflux esophagitis are basal cell hyperplasia, inflammatory cells in the squamous epithelium, and elongation of the lamina propria papillae. Inflammatory infiltrate in lamina propria may comprise neutrophils, eosinophils, and lymphocytes. Together with dilated intercellular spaces and erosion, these signs can now be used as semi-quantitative diagnostic criteria for reflux esophagitis (Table 1).

Many individuals without reflux may show mild epithelial hyperplasia, elongation of the papillae, and occasional eosinophils 2–3 cm proximal to the lower esophageal sphincter as a result of physiologic reflux [42].

**Figure 1:**
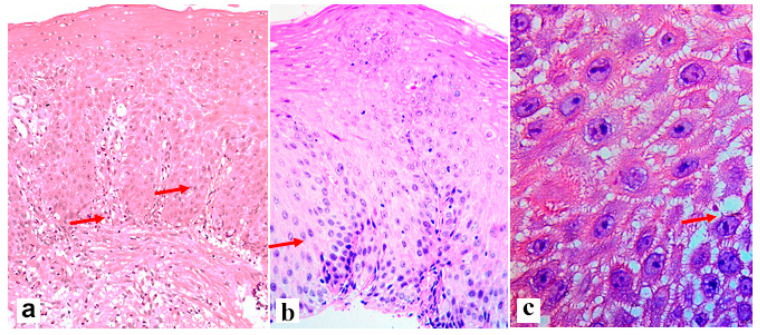
The severity of the histological signs of erosive esophagitis in the edge of the erosive area of a 62-year-old patient with an 8-year history of GERD and endoscopic signs of reflux esophagitis grade C according to LA. (**a**)—Elongation of stromal papillae and basal cell hyperplasia (arrows), (**b**)—basal cell hyperplasia with proliferative acanthosis (arrow), (**c**)—dilated intercellular spaces (arrow). There is a mononuclear inflammatory infiltrate in the lamina propria. Hematoxylin and eosin. (**a**)—×200, (**b**)—×400, (**c**)—×900.

**Figure 2:**
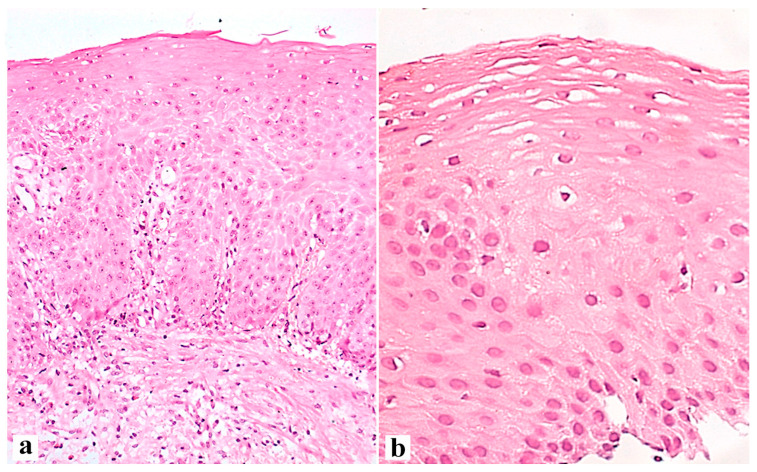
Esophageal mucosa in the area of the erosive defect of a 62-year-old patient with an 8-year history of GERD and endoscopic signs of reflux esophagitis grade C according to LA before (**a**) and 4 weeks after the start of the therapy with PPI and esophagoprotector (**b**). (**a**)—Hyperplasia of the basal layer venular ectasia; the height of the stromal papillae is 60%, dilated intercellular spaces are shown. (**a**)—×200. (**b**)—Weakly expressed basal cell hyperplasia; weak expansion of intracellular contacts is irregular. Hematoxylin and eosin. (**a**)—×200, (**b**)—×400.

**Figure 3:**
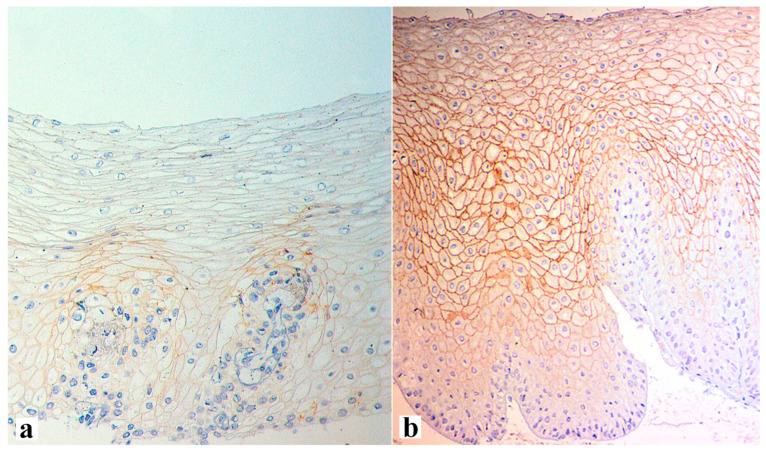
Claudin-1 expression at the edge of the erosive area of a 62-year-old patient with an 8-year history of heartburn and on-demand antacid intake, obesity (BMI 32.5), arterial hypertension, and type-2 diabetes, as well as endoscopic signs of LA grade C reflux esophagitis before (**a**) and after therapy with PPI and esophagoprotector (**b**). Loss of the marker expression in the most epithelium cells of the upper layer (**a**) and the presence of expression over all thicknesses of the epithelial layer after therapy (**b**) are shown. (**a**)—×400, (**b**)—×250.

An illustration of histological signs of reflux esophagitis and a decrease in the expression of the claudin-1 protein in a patient with GERD is shown in Figure 1, Figure 2 and Figure 3.

Thus, the combination of inflammatory processes, oxidative stress, and increased proliferative activity can create a favorable environment for the progression of dysplastic and neoplastic changes in the mucosa, including Barrett’s esophagus and adenocarcinoma [10,45,46].

It is known that the formation of intestinal metaplasia of the esophageal epithelium with subsequent dysplasia and malignant modification is characterized by an increase in proliferation and a decrease in apoptosis in proportion to the progression of pathological changes [47]. Therefore, markers of apoptosis and proliferation can be used to diagnose and assess the prognosis of disease development in patients with GERD.

**Figure 4:**
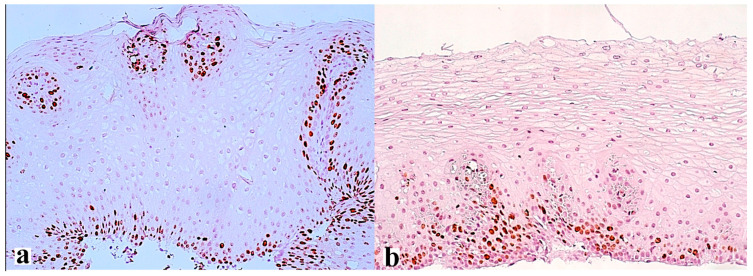
Ki-67 expression in the area of the erosive defect of the esophageal mucosa of a 62-year-old patient with an 8-year history of heartburn and on-demand antacid intake, obesity (BMI 32.5), arterial hypertension, and type-2 diabetes, and endoscopic signs of LA grade C reflux esophagitis before (**a**) and after the treatment (**b**). Increased height of stromal papillae with positive marks in the most basal cells (**a**) and only in individual cells for comparison (**b**). (**a**,**b**)—×200.

Thus, the expression of the Ki-67 protein (Figure 4) is a classic marker of cell proliferation, which is assessed by determining the index of proliferative activity using monoclonal antibodies to Ki-67. This is a short-lived, double-stranded protein that is destroyed within 2 h, and therefore is detected only in tissues with dividing cells in the active phase of the cell cycle throughout its entire length (G1-, S-, G2-, and M -phases). This marker is not found in cells in the G0 phase. Proliferative activity correlates with the severity of inflammation of the esophageal mucosa and increases with the development of metaplasia and dysplasia of the esophageal epithelium. The degree of Ki-67 overexpression correlates with the severity of the inflammatory process in the mucosa of the esophagus [48]. In another study, the same scientists analyzed the expression of Ki-67 in the biopsy specimens of 200 patients with GERD and 35 patients with adenocarcinoma of the esophagus divided into five groups: those with normal squamous epithelium, with the presence of esophagitis, with columnar epithelium without intestinal metaplasia, with columnar epithelium with intestinal metaplasia, and with adenocarcinoma of the esophagus. It was found that Ki-67 expression increased depending on the severity of histopathological changes [49]. In another study, the proportion of Ki-67 positive cells of the intact esophageal mucosa was 4%, which increased to 27.5% in the presence of metaplasia, and to 41.7% in Barrett’s adenocarcinoma [50]. According to other authors, an increase in Ki-67 expression was observed with the development of low-grade dysplasia (up to 14% of labeled cells), up to 73% with high-grade dysplasia, and reached 87% with the development of esophageal adenocarcinoma [51].

MicroRNAs are small non-coding molecules responsible for the regulation of translation and/or degradation of messenger RNAs (mRNAs), increasing or decreasing the synthesis of the proteins they encode, including participating in the transcriptional and post-transcriptional regulation of the expression of tumor suppressor genes and oncogenes [52]. There is evidence of microRNA activation during the formation of Barrett’s esophagus and associated adenocarcinoma in patients with unfavorable GERD [52,53]. The increased production of microRNAs may indicate a high proliferative activity of the tissue, which gives reason to use the molecule as a marker of proliferation. Thus, miRNA-21 expression in patients with Barrett’s esophagus and esophageal adenocarcinoma is 3–5 times higher than in patients with esophageal mucosa without pathological changes [53,54].

The DNA damage marker is the p53 protein encoded by the TP53 gene. In the presence of mutations, the p53 protein accumulates. Detection of the expression of this marker characterizes the stage of the pathological process: 5% indicates the absence of dysplasia, 10–20% indicates low-level dysplasia, 60% indicates high-level dysplasia, and over 70% indicates the presence of adenocarcinoma of the esophagus [55,56]. When DNA is damaged, the p53 protein activates its repair mechanism, or cell apoptosis is triggered when DNA cannot be repaired. When apoptosis genes are blocked or cease to recognize damaged DNA regions, the cell proceeds to uncontrolled division and tumor formation. In the study, 138 patients with symptoms of GERD were divided into four groups depending on the severity of esophagitis according to biopsy data. The overexpression of p53 was 7% in the normal epithelium group, 21.4% in the mild esophagitis group, 52.2% in the moderate esophagitis group, and 60% in the severe esophagitis group. It was found that the overexpression of p53 correlated with the severity of the inflammatory process in the mucosa of the esophagus [48]. In another study by the same research team, p53 expression was analyzed in biopsies of 200 patients with GERD and 35 patients with adenocarcinoma of the esophagus. The patients were divided into five groups: with normal squamous epithelium, with esophagitis, with columnar epithelium without intestinal metaplasia, with columnar epithelium with intestinal metaplasia, and with adenocarcinoma of the esophagus. The study confirmed the direct dependence of p53 expression on the severity of histopathological changes in the mucosa [49]. The possibility of assessing the prognosis of the course of diseases of the esophagus based on the determination of p53 expression remains debatable. On the one hand, a study published in 2013 involving 266 patients showed no relationship between the level of p53 expression and disease prognosis [57]. On the other hand, a number of studies have found that p53 overexpression is associated with low five-year survival, more frequent metastasis in patients with esophageal cancer, and is a marker of poor prognosis [58,59].

## 4. Conclusions

Esophageal mucosal resistance to aggressive reflux molecules under GERD conditions should be considered as one of the most important factors in the formation and progression of reflux esophagitis. Current diagnostic strategies use biopsy in only a few cases: differential diagnosis with other esophagites, as well as in case of complicated disease (such as screening and morphological monitoring of Barrett’s esophagus). At the same time, there are available possibilities for verifying the protective properties of esophageal mucosa by assessing its resistance using modern classification approaches. Immunohistochemical methods can also be used to obtain unique data for the characteristics of reflux-esophagitis and the further development of GERD therapy.

## Figures and Tables

**Table 1:** Proposed histological criteria and score for reflux esophagitis assessment (according to Triantos C. et al., Yerian L. et al., and Savarino E. et al. with changes) [42,43,44].

Criterion	Definition of the Lesions and Their Assessment	Severity Score
1. Basal cell hyperplasia	Measure basal cell layer ^1^ in μm and express as a proportion of total epithelial thickness ^2^	0 (absent < 15%), 1 (15–30%), and 2 (>30%). For Z-line 1 (>20%)
2. Papillary elongation	Measure papillary length in μm and express as a proportion (%) of total epithelial thickness	0 (absent < 50%), 1 (50–75%), and 2 (>75%). For Z-line 1 (>66%)
3. Dilated intercellular spaces	Include irregular round dilations or diffuse widening of the intercellular space ^3^ Small intercellular space = diameter < 1 lymphocyte Large intercellular spaces = diameter ≥ 1 lymphocyte	0 (≤5 small), 1 (≥6 small and ≤5 large), and 2 (≥6 large)
4. Intraepithelial eosinophils	Count cells in the most affected power field ^3^	0 (0 cells in one high-power field), 1 (1–2 cells), 2 (>2 cells)
Combined severity score	Sum of lesion severity scores divided by four (the number of lesions assessed)	
Diagnostic criteria: 0–0.25 normal mucosa, 0.5–0.75 mild esophagitis, ≥1 severe esophagitis		

Biopsies must be taken from the Z-line and at 2–3 cm above it. ^1^—The upper limit of the basal layer is defined as the level above which the nuclei are separated by a distance greater than the nuclear diameter; ^2^—low-power field, objective = 10); ^3^—high-power field, objective = 40).

## Data Availability

Not applicable.
